# The epistemic innocence of motivated delusions ☆

**DOI:** 10.1016/j.concog.2014.10.005

**Published:** 2014-11-06

**Authors:** Lisa Bortolotti

**Affiliations:** University of Birmingham, Edgbaston B15 2TT, United Kingdom

**Keywords:** Delusions, Anosognosia, Erotomania, Reverse Othello syndrome, Motivation, Epistemic status, Anxiety, Wellbeing, Beliefs, Emotions

## Abstract

Delusions are defined as irrational beliefs that compromise good functioning. However, in the empirical literature, delusions have been found to have some psychological benefits. One proposal is that some delusions defuse negative emotions and protect one from low self-esteem by allowing motivational influences on belief formation. In this paper I focus on delusions that have been construed as playing a defensive function (*motivated delusions*) and argue that some of their psychological benefits can convert into epistemic ones. Notwithstanding their epistemic costs, motivated delusions also have potential epistemic benefits for agents who have faced adversities, undergone physical or psychological trauma, or are subject to negative emotions and low self-esteem. To account for the epistemic status of motivated delusions, costly and beneficial at the same time, I introduce the notion of *epistemic innocence*. A delusion is epistemically innocent when adopting it delivers a significant epistemic benefit, and the benefit could not be attained if the delusion were not adopted. The analysis leads to a novel account of the status of delusions by inviting a reflection on the relationship between psychological and epistemic benefits.

## 0 Introduction

In this paper, I ask whether delusions that have been construed as playing a defensive function have epistemic benefits. Defence mechanisms are “a means of nuancing or processing information such that it is rendered less anxiety-provoking” (McKay, Langdon, & Coltheart, 2005, p. 316). Arguably, delusions can prevent loss of self-esteem and help manage strong negative emotions (Butler, 2000; Ramachandran, 1996; Raskin and Sullivan, 1974; Bentall, 1994). The claim that delusions are psychologically adaptive can be made on these grounds, and it was recently discussed in the psychological literature (McKay & Dennett, 2009; McKay & Kinsbourne, 2010; McKay et al., 2005). Without denying that delusions are typically false and irrational, and that they compromise good functioning to a considerable extent, my goal here is to establish whether the psychological benefits attributed to those delusions that have been construed as playing a defensive function can translate into epistemic benefits. Thinking about delusions in terms of potential epistemic benefits leads to a more balanced view of the role of delusions in a person’s cognitive and affective life and invites a reflection on the relevance of contextual factors in epistemic evaluation.

In Section 1, I review the general features of delusions and describe the epistemic features of delusions that can be construed as playing a defensive function (hereafter, *motivated delusions*) and their adverse effects on functioning. In Section 2, I consider arguments for the psychological benefits of Reverse Othello syndrome, erotomania and anosognosia. I also describe the proposal by McKay and Dennett (2009), according to which some false beliefs (*adaptive misbeliefs*) are the result of a mechanism that allows motivational factors to influence belief formation. Can motivated delusions be adaptive misbeliefs? In Section 3, I introduce the notion of *epistemic innocence*. Cognitions are epistemically innocent when, despite their epistemic costs, they carry a significant epistemic benefit (*Epistemic Benefit* condition) that could not be attained otherwise (*No Alternatives* condition). I argue that motivated delusions have the potential for satisfying the two conditions for epistemic innocence and I offer an illustration from anosognosia to support this claim. In Section 4, I suggest that the epistemic innocence potential of motivated delusions highlights the need for a more nuanced evaluation of epistemically costly cognitions and invites a new way of understanding the relationship between psychological and epistemic benefits.

## 1 Motivated delusions

Clinical delusions are symptoms of psychiatric disorders such as schizophrenia, dementia, and delusional disorders. Delusions exemplify failures of rationality and are defined on the basis of surface features that have an epistemic character. Here are some popular definitions:

A false belief based on incorrect inference about external reality that is firmly held despite what almost everyone else believes and despite what constitutes incontrovertible and obvious proof or evidence to the contrary. The belief is not ordinarily accepted by other members of the person’s culture or subculture (i.e., it is not an article of religious faith). When a false belief involves a value judgment, it is regarded as a delusion only when the judgment is so extreme as to defy credibility.

[American Psychiatric Association, DSM-5, 2013, p. 819]

A person is deluded when they have come to hold a particular belief with a degree of firmness that is both utterly unwarranted by the evidence at hand, and that jeopardises their day-to-day functioning.

[McKay et al., 2005, p. 315]

Delusions are generally accepted to be beliefs which (a) are held with great conviction; (b) defy rational counter-argument; (c) and would be dismissed as false or bizarre by members of the same socio-cultural group.

[Gilleen & David, 2005, pp. 5–6]

The definitions above characterise delusions on the basis of their epistemic features, including lack of warrant, fixity, resistance to counterargument, and implausibility. Not all delusions manifest such features to the same extent, and delusions may differ in the way they interact with the person’s other cognitive or affective states.

### 1.1 Types of delusions

Davies, Coltheart, Langdon, and Breen (2001) helpfully distinguish between circumscribed and elaborated delusions. *Circumscribed* delusions are not well integrated with the other beliefs the person has, and the epistemic features that characterise these delusions do not necessarily “spread” to the rest of the person’s belief system. Some of these delusions, so-called “deficit” delusions (McKay & Dennett, 2009), are the result of brain damage or cognitive deterioration, and, even if they were given a psychodynamic interpretation in the past, there is now little room for motivational factors in an account of their formation. Examples are the Capgras delusion (the belief that a loved one has been replaced by an impostor) and mirrored-self misidentification (the belief that there is a stranger in the mirror when one looks at one’s own reflection). Other circumscribed delusions have been construed as playing a defensive function, and motivational factors are sometimes advocated in the explanation of their formation. Such delusions often follow trauma. Examples are the Reverse Othello syndrome (the belief that one’s romantic partner is faithful when she is not) and anosognosia (the denial of illness, for instance the denial that one’s limb is paralysed).

Delusions emerging in the context of schizophrenia can be *systematised* and *elaborated*. They can turn into complex narratives used to explain most of the person’s experience. Examples are the delusion of persecution (the belief that others are threatening and intend to cause harm), the delusion of grandeur (the exaggerated belief in one’s self-worth), and the delusion of reference (the belief that some events are highly significant when they are not). A popular hypothesis is that delusions in schizophrenia are offered as an explanation for the person’s hypersalient experience. Given that hypersalient experiences cause anxiety and distress in the prodromal phase of psychosis, delusions emerge as hypotheses by which the person makes sense of their experiences (Jaspers, 1963; Kapur, 2003; Mishara and Corlett, 2009). Delusions in schizophrenia can put an end to a state of uncertainty that causes anxiety and distress. Sometimes the “need for closure” is discussed in this context (McKay & Kinsbourne, 2010) and it indicates a preference for certainty over uncertainty and for predictability over unpredictability. In addition to satisfying the need to have an explanation as opposed to none, it has been argued that some delusions in schizophrenia can be motivated due to their specific content. Among others, delusions of grandeur and delusions of persecution seem to protect the person from a negative conception of the self and from low self-esteem.

In terms of aetiology, it is plausible that a combination of neurobiological and psycho-social factors (including motivational factors) contribute to the formation of delusions (Bentall, Kinderman, & Kaney, 1994; Davies, 2009; McKay & Kinsbourne, 2010; Roberts, 1992). For instance, Aimola Davies and Davies (2009) argue that a two-factor theory of delusion formation can make sense of most delusions, where the first factor explains where the delusion comes from, and the second factor explains why the delusion is not rejected. The first factor usually consists in an anomalous experience or a neuropsychological deficit. The second factor consists in an impairment of belief evaluation, that is, a problem with the assessment of the evidence for and against the delusional belief. Such a problem may be caused by cognitive impairments, but may also be caused by a motivationally-biased handling of the evidence. When no anomalous experience or deficit can be identified, it is possible that motivation constitutes the first factor, in which case the delusion would emerge as a defence mechanism (McKay et al., 2005).

Here I do not propose to discuss the role of motivation in delusion formation, rather I am interested in evidence for the claim that delusions have psychological benefits, and such evidence is often reviewed in the discussion of different delusion formation theories. For convenience and due to space limitations, I will concentrate on those delusions that have been *explicitly construed as* defence mechanisms in the psychological literature, but I make no assumption about such accounts being the best explanations of how the delusions are formed. For some of the delusions I shall describe, it is possible that a defence mechanism explains the origin of the delusion and its content. For all of these delusions, it is plausible that motivational factors play some role in the maintenance of the delusions. But motivational factors need not play any role in delusion formation for delusions to have psychological benefits.

### 1.2 What is wrong with motivated delusions?

Let me describe some of the characteristics of motivated delusions that amount to epistemic costs and can also lead to impaired functioning. Motivated delusions can be characterised as irrational beliefs, in that they are implausible, they do not accurately represent reality, they do not respond to evidence, and they may not always be consistently reflected in behaviour (Bortolotti, 2009). For instance, people with motivated delusions may be convinced of the truth of the delusion but at the same time exhibit some “covert recognition” that the content of the delusion is false. When they are circumscribed, motivated delusions may conflict with other beliefs the person has, contributing to an overall inconsistent set of beliefs. When they are elaborated and systematised, they are likely to integrate well with a number of other beliefs that also play a defensive function. I will consider three cases of motivated delusions.

An example of a monothematic delusion with a defensive function that emerged as a result of brain damage is the case of Reverse Othello syndrome (Butler, 2000) discussed in some detail by McKay et al. (2005). A man, BX, delusionally believed that he was in a happy relationship, when in fact his partner had left him.

Butler’s patient was a talented musician who had sustained severe head injuries in a car accident. The accident left him quadriplegic, unable to speak without reliance on an electronic communicator. One year after his injury, the patient developed a delusional system that revolved around the continuing fidelity of his partner (who had in fact severed all contact with him soon after his accident). The patient became convinced that he and his former partner had recently married, and he was eager to persuade others that he now felt sexually fulfilled.

[McKay et al., 2005, p. 313]

BX’s belief in the fidelity of his previous partner and the continued success of his relationship was very resistant to counterevidence. BX believed that his relationship was going from strength to strength for a few months, even though his former partner did not want to communicate with him and was in a relationship with someone else (Butler, 2000, p. 86).

The Reverse Othello syndrome can be seen as a special case of erotomania. In erotomania, a person comes to believe that another person, often of a perceived higher status (e.g., a teacher, an older or more successful person, a celebrity), is in love with her when there is no evidence in support of that belief. Here is the case of a young woman, LT, who started behaving strangely when she became obsessed with the idea that a fellow student was in love with her although the two had never spoken to each other.

Her conversation, when unrelated to her delusional process, was rational, coherent, appropriate, and relevant. […] When speaking of the delusional process, she went into great detail, explaining the messages she received from her fantasied lover, signs which she received on TV, from the colors of dresses, license plates on cars, and from several other sources. She saw all of this as proof of the fact that the young man was in love with her and was planning to marry her.

[Jordan & Howe, 1980, p. 983]

Numerous attempts to offer LT evidence that her belief was false failed: after two and a half years after the delusion emerged, the alleged lover was asked to talk to LT on the phone, following a suggestion by LT’s mother. He told LT that he did not have any intention to marry her and that he could barely remember who she was. But LT was convinced that her mother had arranged for her to talk to another man and did not abandon her delusion.

Motivated delusions are also found in the context of anosognosia, the denial of illness (most commonly, the denial that a limb is paralysed). Delusions take the form of: “I am moving my arm”, when the arm cannot move; or “I can climb stairs but I am a little slow” when a leg is paralysed. Anosognosia has been considered as a pathology of belief: “There is a mismatch between the patient’s estimate of his or her abilities and the reality of the impairment” (Aimola Davies, Davies, Ogden, Smithson, & White, 2009, p. 188). In anosognosia, people deny evidence supporting the fact that they are impaired. Here is the case of a patient with anosognosia for hemiplegia:

[A]sked to clap the hands, [she] lifted her right hand and put it in the position of clapping, perfectly aligned with the trunk midline, moving it as if it was clapped against the left hand. She appeared perfectly satisfied with her performance, never admitting that the left arm did not participate in the action. This despite the fact that the patient could see that the left hand did not clap against the right hand and the typical sound of clapping was not heard.

[Berti, Spinazzola, Pia, & Rabuffetti, 1993, p. 164]

It is not clear to what extent people with anosognosia are unaware of their impairment, given that, on occasion, they seem to implicitly acknowledge it. For instance, a person with a paralysed leg might deny paralysis but at the same time acknowledge that she cannot climb stairs properly. She might even provide a confabulatory explanation of her poor performance, saying that it is due to tiredness or to arthritis (Ramachandran, 1995, p. 23).

The delusions I have described are not just implausible and irresponsive to evidence but they have an adverse effect on wellbeing and interfere with interpersonal relationships. The person reporting the delusion stops being regarded as a trust-worthy source of information about the topic of the delusion and may be socially sanctioned or excluded for that reason. Relationships with family members and with healthcare professionals may be strained as a result of the absence of a “shared reality” (Fotopoulou, 2008, p. 546) and common goals.

In many cases of erotomania (Lovett-Doust & Christie, 1978), the person develops an obsession with the delusional theme, loses interest in her family and friends, gives up her daily activities, and becomes isolated as a result. Anosognosia can have negative effects on people’s health. For instance, it can interfere with therapy and rehabilitation (Fotopoulou, 2008, p. 554). If the person does not acknowledge an impairment due to a recent trauma, she will not understand the need to engage in rehabilitation; indeed it has been found that anosognosia is “an inhibitory factor hampering rehabilitation” (Maeshima et al., 1997, p. 691).

## 2 Delusion as psychologically adaptive

Considerations about the epistemic costs of delusions and their adverse effects on functioning seem to rule out the possibility that delusions have any benefits. However, it has been argued that the adoption of a motivated delusion helps manage overwhelmingly negative emotions that would otherwise lead to depression and protects against negative self-conceptions that would otherwise lead to low self-esteem. In the light of these arguments, the relationship between delusions and wellbeing appears more complex than one might have expected. For instance, Lansky (1977, p. 21) writes that delusion “is restitutive, ameliorating anxieties by altering the construction of reality”.

### 2.1 Motivated delusions in context

In the case of Reverse Othello syndrome I mentioned earlier, the delusion seemed to protect BX from the undesirable truth that his romantic partner had left him while he was coping with the consequences of permanent disability. Butler, who first reported on this case, describes the period preceding the report of the delusion:

[H]is communicative responses initially indicated considerable insight and the beginning of an intense emotional response to a massive disability and a fracturing of his interpersonal relationships.

[Butler, 2000, p. 87]

Gradually, in the year following his injury, BX developed the delusion that his former romantic partner was still in a successful relationship with him, and also that they had recently married. While still in hospital, he often asked to go home so that he could see his wife. Butler argues that the delusion relieved the sense of loss that BX was feeling at the time.

[A]ppearance [of delusions] may mark an adaptive attempt to regain intrapsychic coherence and to confer meaning on otherwise catastrophic loss or emptiness.

[Butler, 2000, p. 90]

As gradually as it had appeared, BX’s delusional system dissolved, and by the end of the process BX realised that his former partner had moved on, was not married to him, and had no intention to go back to him. No other delusions or psychotic symptoms were observed. This happened roughly at the time when BX had completed his physical rehabilitation and was ready to return home.

Butler does not advocate a purely motivational explanation for BX’s delusion, but simply argues that different factors might have been at play, and that a psychological defence against depression contributed to the fixity and elaboration of BX’s delusional system. Independent of the role of motivational factors in the formation or maintenance of delusions, Butler makes a case for the presence of psychological benefits. The delusion kept BX’s depression at bay at a very critical time. Acknowledging the end of his romantic relationship might have been disastrous at a time when he was coping with the realisation of his new disability and its effects on his life.

Erotomania more generally has also been described as an adaptive response (Raskin & Sullivan, 1974)), and it is significant that it often develops following a physical/psychological trauma or adverse circumstances. In many of the case studies of erotomania reported in the literature, the delusion seems to affect people who have a history of depression and feel lonely or under-appreciated (Hollender & Callahan, 1975). Consider LT, the young woman who developed erotomania and whose case was reported by Jordan and Howe (1980). Her background fits with the general profile described:

The patient has a twin sister, who at that time was a junior in college, and a younger sister, who was two years old at the time of the onset of this disorder. In describing L.T.’s twin sister, Mrs. T. indicated that she was outgoing, friendly, and though somewhat reserved, maintained close relationships with people of both sexes. The mother stated that the patient in contrast had always been a quiet and rather inhibited child. She was much more reserved than her popular sister and dated infrequently. She was also described as being studious, an avid reader, highly moralistic, and a loner. Moreover, she tended to be somewhat suspicious and mistrustful. Her limited heterosexual experiences were characterized as being very short-lived. According to the mother, one such relationship had just recently ended abruptly and she related that the patient appeared rather emotionally distraught by this.

[Jordan & Howe, 1980, pp. 982–3]

In many cases of erotomania (Lovett-Doust & Christie, 1978, p. 105), there are identifiable “pharmacological, metabolic, and physiological and structural causes” (including injection of cortisone, alcoholism, meningioma, ingestion of contraceptive pills), but also “psychological and situational triggers”. People experience loneliness and loss prior to adopting the delusion, and in some cases the emergence of the delusion follows a traumatic event (e.g., the discovery of one’s partner’s infidelity, the death of a loved one, or the birth of a child then given up for adoption). This seems to suggest that the delusion plays a defensive function in that it compensates for loss or protects one from low self-esteem.

In anosognosia, the connection between trauma and delusion is more explicit. The person refuses to acknowledge a serious impairment as a result of trauma or illness and, also often fails to recognise its implications (although the denial of the impairment and the failure to acknowledge its implications can dissociate, see Aimola Davies et al., 2009). Delusions occurring in anosognosia can be seen as playing a defensive function, but purely motivational accounts of anosognosia have been strongly criticised for failing to account for the fact that anosognosia is much more likely to emerge when the right parietal lobe is damaged, and that the denial seems to be “domain-specific”: the person may deny one impairment and acknowledge another. Ramachandran describes a patient who would go to great lengths to deny the paralysis of her limb but happily admitted to having diabetes (Ramachandran, 1995, pp. 23–24).

To explain these phenomena, popular accounts of anosognosia have attempted to combine neuropsychological and motivational factors (Aimola Davies et al., 2009; Ramachandran, 1996). Ramachandran advances the hypothesis that the behaviours that give rise to delusions in this context are an exaggeration of normal defence mechanisms that have an adaptive function. Denying change can sometimes be instrumental to preserving a coherent system of beliefs and behaving in a stable and predictable manner (Ramachandran, 1996). The psychological advantages are not necessarily cashed out in terms of the preservation of the concept of the self *as healthy*, but in terms of the preservation of the concept of the present self *as coherent* with that of the past self. Fotopoulou observes the same phenomenon in people with memory impairments and anosognosia who do not seem to update personal information:

[P]atients may need to highlight their continuity and coherence with their past selves and may not be able to understand or deal with the loss of their previous family and social role.

[Fotopoulou, 2008, p. 560]

Aimola Davies and Davies (2009) report positive and negative effects of anosognosia on wellbeing, suggesting that there could be a role for motivational factors in the explanation of anosognosia. As I mentioned in Section 1.2, anosognosia has negative effects, as people who do not acknowledge the illness or impairment may be slow in seeking treatment and unmotivated to engage in rehabilitation. But after the initial stages of illness, anosognosia is associated with fewer negative emotions and reduced anxiety.

### 2.2 Delusions as a shear pin

According to the “shear-pin” account developed by McKay and Dennett (2009), some false beliefs that help manage negative emotions and avoid low self-esteem and depression can count as *psychologically adaptive*. McKay and Dennett suggest that, in situations of extreme stress, motivational influences are allowed to intervene in the process of belief evaluation, causing a *breakage*. Although the breakage is bad news epistemically, as the result is that people come to believe what they desire to be true and not what they have evidence for, it is not an evolutionary “mistake”, rather it is designed to avoid breakages that would have worse consequences for the person’s self-esteem and wellbeing.

What might count as a doxastic analogue of shear pin breakage? We envision doxastic shear pins as components of belief evaluation machinery that are “designed” to break in situations of extreme psychological stress (analogous to the mechanical overload that breaks a shear pin or the power surge that blows a fuse). Perhaps the normal function (both normatively and statistically construed) of such components would be to constrain the influence of motivational processes on belief formation. Breakage of such components, therefore, might permit the formation and maintenance of comforting misbeliefs – beliefs that would ordinarily be rejected as ungrounded, but that would facilitate the negotiation of overwhelming circumstances (perhaps by enabling the management of powerful negative emotions) and that would thus be adaptive in such extraordinary circumstances.

[McKay & Dennett, 2009, p. 501]

Could motivated delusions be *adaptive misbeliefs*? The mechanism that inhibits motivational influences on belief evaluation would be compromised, and as a result of this motivated delusions would emerge, making negative emotions easier to manage and depression less likely to ensue. McKay and Dennett consider the possibility that some delusions count as adaptive misbeliefs, but interestingly argue that *the extent to which* desires are allowed to influence belief formation in the case of delusions is pathological. Delusions are the result of the *maladaptive* version of a psychologically adaptive mechanism.

Delusions may be produced by extreme versions of systems that have evolved in accordance with error management principles, that is, evolved so as to exploit recurrent cost asymmetries. As extreme versions, however, there is every chance that such systems manage errors in a maladaptive fashion.

[McKay & Dennett, 2009, p. 502]

More needs to be said about the precise nature of the advantage that adaptive misbeliefs may have, but shear-pin accounts are helpful in providing a framework for the potential psychological benefits of motivated delusions. First, in the shear-pin account, the situation in which adaptive misbeliefs emerge is already seriously compromised. The premise is that the person is already experiencing high levels of distress, and can come to more serious harm unless her negative emotions are managed. Thus, the benefit here amounts to the prevention of more serious harm than the one the person is already experiencing. In other words, the adaptive misbelief is equivalent to an emergency response. McKay and Dennett talk about the “extraordinary circumstances” in which motivational influences on belief are not just tolerated but desirable, and argue that such influences are not accidental but designed. I am going to suggest that a careful consideration of the circumstances in which beliefs are adopted should play a role in establishing not just whether they have psychological benefits, but also what their epistemic status is.

Second, the phrase “adaptive misbelief” and the general description of the shear-pin mechanism may be taken to suggest that there is almost an inverse correlation between psychological and epistemic benefits. The more distant the belief is from a bleak reality, the more psychologically adaptive it is. This is not, however, what McKay and Dennett have in mind. Indeed, a possible explanation for the difference between motivated delusions and non-delusional adaptive misbeliefs is that delusions are ultimately *maladaptive* because, in the case of delusions, motivational influences affect beliefs to an extent that compromises their overall plausibility and makes them impervious to counterevidence. These epistemic costs are likely to bring also psychological costs, and thus delusions may turn out to have greater psychological costs than benefits. I am going to suggest that we should resist a trade-off view of the relationship between psychological and epistemic benefits. There are reasons to believe that it is psychologically beneficial to have beliefs that are constrained by reality, and that managing negative emotions, relieving anxiety, and protecting self-esteem have epistemically positive consequences.

## 3 Epistemic innocence

What is it to be epistemically innocent? Innocence is sometimes cashed out in terms of a person being ‘free from faults’ or ‘free from sins’. This is not the sense of innocence I am advocating here. Ideally, agents would have beliefs that are true and that are supported by, and responsive to, the evidence available to them. But human agents have limited cognitive capacities, and beliefs that are false and badly supported by, or irresponsive to, the evidence are a common occurrence. It is tempting to dismiss epistemically costly cognitions altogether. But sometimes an epistemically costly cognition can also have positive epistemic features. When these epistemic benefits are significant and could not be attained in other ways, then the cognition may gain some sort of *innocence*.

The notion of innocence I have in mind is used in the legal context of justification defence. In general, an *innocence defence* applies to someone who is not deemed liable for an act that appears to be wrongful. Innocence defence can be due to *excuse* or *justification*. An excuse defence applies when there is no criminal intent. A justification defence applies when the act does not constitute an offence in the given circumstances because it prevents greater harm from occurring. Here is an example of a justification defence: Ann injures Ben and by doing so she stops him from detonating a bomb (Greenawalt, 1986, p. 89). It is morally and legally objectionable to cause injury to another, but in the specific circumstances in which Ann injures Ben her action has some significant benefits as it prevents a greater harm from happening. Moreover, other ways of stopping Ben, such as talking him out of detonating the bomb, may be not available at the time. Ann is acquitted because what she did is not wrongful: it is an acceptable response to an emergency.

My purpose here is to apply this notion of innocence to the domain of epistemic evaluation. In some contexts, an epistemically costly cognition (say, a false belief) may help avoid bad epistemic consequences, and thus qualifies as an acceptable response to an emergency.^1^ For instance, a delusion is epistemically innocent if adopting it delivers a significant epistemic benefit that could not be obtained otherwise.

I propose the following two conditions for epistemic innocence:

*Epistemic Benefit*: The delusional belief confers a significant epistemic benefit to an agent at the time of its adoption.
*No Alternatives*: Other beliefs that would confer the same benefit are not available to that agent at that time.^2^

The exact formulation of the conditions for epistemic innocence will vary depending on one’s epistemological commitments. Let us consider the Epistemic Benefit condition first. The epistemic benefits of having a cognition may include maximising the acquisition and retention of true beliefs (for a veritist), promoting intellectual virtues (for a virtue epistemologist), or avoiding epistemic blame (for a deontologist). In line with a broadly consequentialist understanding of epistemic value, I shall argue in Section 3.1 that the adoption of a delusional belief can support the agent’s *epistemic functionality* that would otherwise be compromised by overwhelming negative emotions and low self-esteem. I take epistemic functionality to be the capacity to perform well epistemically by acquiring true beliefs/knowledge or exercising intellectual virtues.

Now let us consider the No Alternatives condition. Different notions and degrees of unavailability can explain the failure to adopt a less epistemically costly cognition. This variety may be due to the nature of the limitations that the agent experiences in the relevant context, ranging from standard reasoning limitations to deficits affecting perception, inference, or memory in clinical settings (see Sullivan-Bissett (2015) for a useful taxonomy of relevant types of unavailability). In short, there may be no alternative to adopting a delusional belief because evidence that would lend support to less epistemically costly beliefs is not available or cannot be weighed up due to a bias or deficit. I shall review these options in Section 3.2.

### 3.1 Meeting the Epistemic Benefit condition

The epistemic benefits of motivated delusions are mediated by their psychological benefits. We saw in Section 2 that the adoption of a delusional belief can be psychologically adaptive. In the shear-pin account of the benefits of delusions, the very fact that people believe a more positive version of reality (e.g., “I am now severely disabled, but my girlfriend still loves me”) than the one they have evidence for allows them to manage negative feelings that could become overwhelming, preserve self-esteem, and overcome anxiety and stress. The benefits in question amount to the delusion preventing a serious epistemic harm from occurring.

McKay and Dennett focus on the effects of adaptive misbeliefs on *wellbeing*. The point of allowing motivational factors to influence belief evaluation is to make the person *feel better* about herself and her situation. But if a belief helps manage negative emotions, protect self-esteem, and relieve anxiety and stress, it will have positive effects not just on the agent’s wellbeing but also on her capacity to function well epistemically (what I called “epistemic functionality”). By having the belief, a person will be more likely to engage with her surrounding physical and social environment in a way that is conducive to epistemic achievements. Consequences of stress and anxiety include lack of concentration, irritability, social isolation, and emotional disturbances. These in turn negatively affect socialisation, making interaction with other people less frequent and less conducive to useful feedback on existing beliefs, and to the fruitful exchange of relevant information. Due to reduced socialisation and engagement, the acquisition and retention of knowledge is compromised and intellectual virtues are not exercised.

There is at least one problem with considering relief from stress and anxiety as an indirect source of epistemic advantages for motivated delusions. The delusion may bring relief at the time when it is adopted, due to the person being already in an epistemically compromised situation, but it often *increases* rather than reduces stress and anxiety when it is maintained in the face of conflicting evidence and challenges from third parties. Stress and anxiety no longer come from the negative emotions associated with trauma or loss (“I’m paralysed”, “My girlfriend left me”, “Nobody loves me”, etc.), but from the fact that the content of the delusion can clash with aspects of the person’s experience, conflict with other things she believes or feels, and alienate other people. For all of these reasons, anxiety and depression do not always lessen after a delusion is adopted, they can also heighten. This is particularly true of delusions with negative content that are correlated with higher depression and lower self-esteem (e.g., Smith et al., 2006). Thus, the adoption of a delusional belief may be beneficial because it prevents the occurrence of a disastrous epistemic breakdown, but its benefits are unlikely to outlive the prevention of the breakdown. It should also be kept in mind that acknowledging that motivated delusions can have some epistemic benefits is not equivalent to claiming that their epistemic benefits outweigh their epistemic costs. My claim is relatively modest: when they are adopted delusions can be epistemically *innocent*, as opposed to epistemically justified, or epistemically good overall.

The obvious question is why the person does not adopt a belief that has the same epistemic benefits as the delusional one but fewer costs. One suggestion emerging from the empirical literature is that, in the “extraordinary circumstances” in which the agent finds herself, no other belief with the relevant characteristics is available.

### 3.2 Meeting the No Alternatives condition

I have reviewed some of the literature suggesting that motivated delusions can help manage negative emotions and avoid low self-esteem because they function as a defence mechanism against the psychological effects of previous adversities or recent trauma. This may be sufficient to claim that, if relief from anxiety and distress benefits agents by supporting their epistemic functionality, then motivated delusions can be thought of as epistemically beneficial in the circumstances. This claim, though, does not amount to an epistemic *defence* of motivated delusions. In order for delusions to be epistemically innocent, we also need to establish that less epistemically costly beliefs could not deliver the same epistemic benefits. This is analogous to the legal case: for Ann to be found innocent of injuring Ben, the court needs to be convinced that there was no other way for her to stop him from detonating the bomb.

How to characterise the availability of alternative beliefs is an issue which deserves greater attention than I can give it here. To make a start, I shall review how the unavailability of alternative beliefs has been assessed with respect to delusions emerging in the context of anosognosia. There are two main obstacles to adopting a non-delusional belief (such as “I am paralysed”): (1) evidence for the truth of the non-delusional belief is not available; and (2) evidence for the truth of the non-delusional belief is available but the agent’s capacity for evaluating competing hypotheses is compromised, and thus the evidence is not taken into account.

To start with option (1), is evidence for their impairment available to people with anosognosia who deny their impairment? The possibility that people may be covertly aware of their impairments has been discussed widely in the empirical and philosophical literature (see, for instance, Fotopoulou, Pernigo, Maeda, Rudd, & Kopelman, 2010; Levy, 2008). Ramachandran (1995) found that people with anosognosia for left hemiplegia who are asked to choose between unimanual tasks (tasks that they can perform by using just one hand) and bimanual tasks (tasks that they can perform only if they use both hands) consistently choose bimanual ones because the expected rewards are greater. Controls (people with paralysis but no anosognosia) choose the unimanual tasks instead. This seems to suggest that people with anosognosia genuinely believe that they can succeed in those tasks. Given these results, there is no reason to suppose that evidence of their impairment is available to them.

Similar conclusions can be drawn from the use of a virtual reality box, where the person is fooled into seeing her (paralysed) arm moving by following the experimenter’s instructions (in reality, the moving arm belongs to someone else). If people were aware of their paralysis, they would show (verbal and non-verbal) signs of surprise, but this is not the case.

[F]ar from being a mere façade-like condition that leaves room for traces of insight to leek through, anosognosia runs deep.

[Ramachandran, 1995, p. 31]

With respect to option (2), some cases of anosognosia show that the person can be made temporarily aware of her impairment (via vestibular stimulation), but lacks the capacity to integrate the information in her overall conception of herself (Ramachandran, 1995, p. 36). As we saw earlier, Ramachandran explains the formation of the delusions in terms of the need to preserve a coherent sense of self. Usually, the left hemisphere produces confabulatory explanations aimed at preserving the *status quo*, but the right hemisphere detects an anomaly between the hypotheses generated by the left hemisphere and reality. So, it forces a revision of the belief system. In people with anosognosia, this discrepancy detector in the right hemisphere no longer works, and the belief system fails to update.

For Aimola Davies and Davies (2009), it is both true that the person has no *direct* evidence of the impairment and that she cannot use the *other available* evidence to revise her belief that she is healthy. Indeed, the formation of anosognosia is explained by the authors in terms of two factors: (1) the person’s motoric failure does not make itself known to the person via direct experience due to neglect, loss of proprioception, or specific problems of integration and memory; and (2) the person cannot use other available evidence of motoric failure due to problems with working memory and executive function. This analysis strongly suggests that evidence for the belief that there is an impairment is not usually available to people with anosognosia: they cannot learn about their impairment from their own experience (due to a neuropsychological deficit that constitutes factor one), and they cannot use other evidence to come to the conclusion that they are impaired (due to their compromised capacity to evaluate competing hypotheses that constitutes factor two).

Even if evidence for non-delusional beliefs were available to people with anosognosia, such beliefs would probably fail to support the agent’s epistemic functionality to the same extent as the delusional beliefs. A more plausible belief (e.g., “I am paralysed”) may not be as well placed as the delusional one to play a defensive function, in terms of preserving a coherent and positive self and defusing the negative emotions caused by trauma and disability. Given that the non-delusional beliefs lack such psychological benefits, they may also lack the epistemic benefits associated with them.

## 4 Conclusions and implications

In this paper I have argued that the psychological benefits of motivated delusions can convert into epistemic ones. Motivated delusions have the potential for epistemic innocence, where epistemic innocence is characterised as the epistemic status of those cognitions that have obvious epistemic costs but also have a significant epistemic benefit that would be otherwise unattainable. Those delusions that allow the agent to manage negative emotions and avoid low self-esteem are also likely to support the agent’s epistemic functionality. Moreover, when they are adopted, motivated delusions may be the only beliefs supporting epistemic functionality that are available to agents distressed by the consequences of previous adversities, recent trauma or loss.

When we think about epistemically costly cognitions that may have psychological benefits, such as self-deception, positive illusions, confabulatory narratives, and distorted memories, we usually think in terms of there being a trade-off. Believing something false or putting a positive spin on a past event can make us feel better, but it leads us further away from the truth. Thus, it may increase wellbeing, but it is not epistemically good. The case for the potential epistemic innocence of motivated delusions puts some pressure on the trade-off view. It would be misleading to believe that motivated delusions provide anxiety-relief and protect self-esteem by compromising access to the truth. Rather, in the picture I have sketched, delusional beliefs are adopted at a time when access to the truth is already compromised by the effects of trauma or previous adversities, and it would be further compromised unless negative emotions were effectively managed. As a temporary response to an emergency, motivated delusions play a useful epistemic function.

These considerations obviously apply to other epistemically costly cognitions. In particular, everyday self-deception and motivated delusions seems to have a very similar shear-pin function in that they are the result of a mechanism that lets desires shape beliefs. In so far as motivational influences on belief formation relieve anxiety and stress, everyday self-deception and motivated delusions can carry some benefits by supporting the agent’s epistemic functionality. But different from self-deception, motivated delusions may invite a *radical* embellishment of the agent’s reality and create tension both within an agent’s belief system and between the agent and other agents, thereby causing inconsistencies, social isolation, and withdrawal. Thus motivated delusions are more likely to become maladaptive (as opposed to adaptive) instances of misbelief than everyday self-deception. On the other hand, the person with everyday self-deception may have more alternative hypotheses available to her than the person who ends up endorsing a motivated delusion, if we suppose the former is not subject to perceptual abnormalities or reasoning impairments to the same extent as the latter. Thus, it may be harder to argue for the epistemic innocence of non-clinical self-deception.

The case of motivated delusions and its analogies and disanalogies with self-deception illustrate perfectly the limitations of the trade-off view: some of the psychological benefits attributed to delusions carry significant epistemic benefits that it would be unwise to neglect. It may seem that wellbeing is safeguarded at the expense of truth when the delusional belief is adopted, but a reflection on the effects of adopting the delusion suggests that safeguarding wellbeing and promoting epistemic functionality go hand in hand.

In the case of Reverse Othello syndrome described earlier, the clinical team decided not to challenge the delusion after they realised that there were no other psychotic symptoms and the delusion was playing a defensive function.

Persistent attempts […] to challenge B.X.’s delusional beliefs were unsuccessful and usually led him to become tearful and agitated.

[Butler, 2000, p. 87]

It was concluded that B.X.’s fantasy system functioned to protect him from the consequences of massive narcissistic injury and attendant depressive overwhelm. All members of the treating team were instructed not to aggressively B.X.’s delusional beliefs but were also cautioned not to become complicit in his elaboration of them.

[Butler, 2000, p. 88]

Similarly, Fotopoulou observes that challenging delusions in a person with anosognosia can prove ineffective and psychologically disruptive:

[RM’s] engagement in rehabilitation activities was initially very poor as he was not motivated and required constant prompting and supervision. Attempts to contradict his anosognosia and increase his motivation were often ineffective as RM immediately provided a series of confabulations to support his alleged abilities and he was particularly sensitive to poor performance and negative feedback.

[Fotopoulou, 2008, p. 554]

The excerpts above make a similar point in different contexts (Reverse Othello syndrome and anosognosia): if the delusional belief provides some psychological benefit and the benefit is not available via any other belief that the person would accept at that time, then challenging the delusion is a bad idea. A clinical team might decide not to challenge a delusion if they think that challenging an agent is going to be ineffective or disruptive, or if there is a high risk of depression ensuing from the agent’s insight into her mental illness.

My discussion suggests that, in these contexts, challenging the delusion might not be advisable from an epistemic point of view either. At the critical stage, motivated delusions may serve a useful epistemic function, allowing the agent to overcome negative feelings or low self-esteem that would prevent her from exercising her epistemic functionality.

## Footnotes

1 In the analogy I draw between innocent acts and innocent cognitions it does not matter whether the act or the cognition counts as voluntary as here I focus on the consequences of the acts and not the intentions of the agent.

2 I consider only beliefs or belief-like states as relevant alternatives here as I am interested in the epistemic evaluation of delusions, that is, how they fare as beliefs or belief-like states. I leave it open that the benefit the delusion confers at the time of its adoption could be gained by other means.
